# Feasibility of remote digital monitoring using wireless Bluetooth monitors, the Smart Angel™ app and an original web platform for patients following outpatient surgery: a prospective observational pilot study

**DOI:** 10.1186/s12871-020-01178-5

**Published:** 2020-10-08

**Authors:** Thierry Chevallier, Gautier Buzancais, Bob-Valéry Occean, Pierre Rataboul, Christophe Boisson, Natacha Simon, Ariane Lannelongue, Noémie Chaniaud, Yann Gricourt, Jean-Yves Lefrant, Philippe Cuvillon

**Affiliations:** 1 Department of Biostatistics, Epidemiology, Public Health and and Methodological innovation (BESPIM), Nîmes University Hospital, University Montpellier 1, Montpellier, France; 2 Staff anesthesiologists, Department of Anesthesiology and Pain Management, Centre Hospitalo-Universitaire (CHU) Carémeau, Place du Professeur Debré, Nîmes, and Montpellier University 1, Montpellier, France; 3 UR 7273 CRP-CPO, Université Picardie Jules Verne, Chemin du Thil, 80000 Amiens, France

**Keywords:** Ambulatory surgery, Remote monitoring, Device, Usability

## Abstract

**Background:**

Remote monitoring of mean arterial blood pressure (MAP), heart rate (HR) or oxygen saturation (SpO_2_) remains a challenge in outpatient surgery. This study evaluates a new digital technology (Smart Angel™) for remotely monitoring hemodynamic data in real time: data transmitted from the patient’s home to a central server, using a dedicated web-based software package.

**Methods:**

Adults scheduled for elective outpatient surgery were prospectively enrolled. In the first 5 postoperative days, patients completed a self-report questionnaire (pain, comfort, nausea, vomiting) and recorded SpO_2_, HR and MAP via two wireless Bluetooth monitors connected to a 4G tablet to transmit the data to a website, in real time, using Smart Angel™ software. Before transmission to the website, these data were also self-reported by the patient on a paper form. The primary outcome was the proportion of variables (self-monitored physiological data + questionnaire scores) correctly transmitted to the hospital via the system compared with the paper version.

On Day 5, a system usability scale survey (SUS score 1–100) was also attributed.

**Results:**

From May 2018 to September 2018, data were available for 29 out of 30 patients enrolled (1 patient was not discharged from hospital after surgery). The remote monitoring technology recorded 2038 data items (62%) compared with 2656 (82%) items recorded on the paper form (*p* = 0.001). The most common errors with the remote technology were software malfunctioning when starting the MAP monitor and malfunctioning between the tablet and the Bluetooth monitor. No serious adverse events were noted. The SUS score for the system was 85 (68–93) for 26 patients.

**Conclusion:**

This work evaluates the ability of a pilot system for monitoring remote physiological data using digital technology after ambulatory surgery and highlights the digital limitations of this technology. Technological improvements are required to reduce malfunctioning (4G access, transmission between apps).

**Trial registration:**

ClinicalTrials.gov (NCT03464721) (March 8, 2018).

## Background

Ambulatory procedures have become a standard of care for all types of surgery, including more and more invasive or complex surgery (abdominal, gynaecological coelioscopic or robotic approaches, hip or knee arthroplasties etc.) [1, 2]. However, safety and adverse events with these procedures remain debated in the literature with regard to potential medical or surgical complications at home [3–7]. Recently, digital technologies have been proposed to remotely monitor outpatients at home and in hospital in order to detect patients with early signs of disease progression or deterioration [8–10].

In ambulatory surgery, patients would normally inform institutions about their perceived condition at home through a text message survey (mobile phone application) or e-mail [11]. Previous studies have described the effect of patients reporting their postoperative recovery after day surgery [11]. Web-based systems collecting alerts, managing and analysing patient-reported outcomes have been added to provide more valuable feedback [12]. The main limitation of these systems is the absence of remote data on physical parameters such as heart or respiratory rates, oxygen saturation and blood pressure. This appears to be a major limitation although several studies have demonstrated that remote monitoring of physiological parameters can significantly reduce morbidity or mortality over the perioperative period [13, 14].

**Fig. 1:**
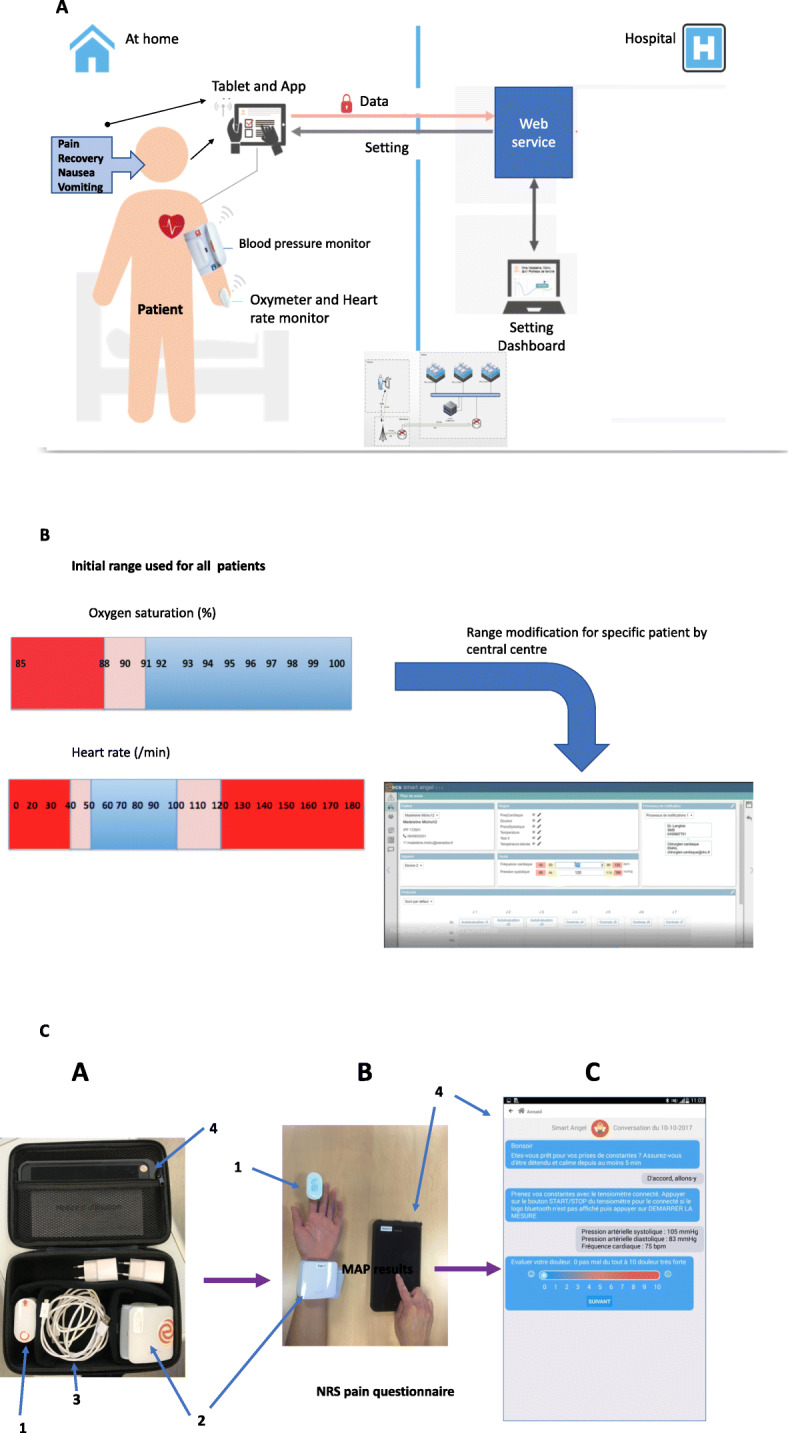
(All illustrations and images provided by the author and never published elsewhere): **a**: System overview: Data are transmitted from the patient’s home to a central server, using a dedicated web-based software package. The data are subsequently processed and presented to health care workers at the hospital. **b**: Range of MAP, SpO_2_, HR for the remote monitoring. **c**: Overview of app and monitor: remote wireless monitoring (**a**), patient with monitors (**b**) and tablet screen (**c**) (Illustration provided by the author and never published elsewhere). **a**: Monitor, tablet and bag. 1: Heart rate and SpO^2^ monitor. 2: MAP monitor. 3: Cables for USB connection or battery. 4: Tablet. 5: Dedicated briefcase. **b**: Connected monitor positioned by the patients themselves and tablet used by patients. **c**: App screen and questionnaires (showing pain on the NRS)

Using a wireless Bluetooth monitor, heart and respiratory rate (HR, RR), mean arterial blood pressure (MAP) or oxygen saturation (SpO_2_) can be recorded via a tablet or smartphone that transmits data from remote monitoring to a web service (central server) (Fig. 1a). Using algorithms and a dashboard, the centre can automatically filter data so that nurses or physicians can focus on patients with early warning signs. Smart Angel™ (Evolucare, France) is a new digital technology for remotely monitoring patients at home using both text messages (self-report questionnaires) and wireless Bluetooth monitors (SpO_2_, HR and MAP). The patient can thus evaluate pain relief on a numerical rating scale (NRS), comfort and adverse events (nausea, vomiting) via self-report questionnaires. The Smart Angel system is initialized at the ambulatory centre before discharge (login) and continued at home by patients using a specific application on a dedicated tablet used to record their self-assessment and start the wireless Bluetooth monitor. Remote data are collected three times a day (Fig. 1a, b, c). This study represents the first stage in testing the device on patients in real-life situations to evaluate its technological capacities and usability by the patient, before applying and testing this technology in current care.

Using the Smart Angel™ app (Evolucare, France), we hypothesized that this system would be able to record and transmit seven variables (pain, quality of recovery, nausea, vomiting, HR, MAP and SpO_2_) for the first 5 days following ambulatory surgery. Before transmission to the website, these data were also reported by the patient on a paper form.

The primary outcome was the proportion of clinical variables (self-monitored physiological data + questionnaire scores) transmitted correctly to the hospital via the IT system. This proportion was also compared with the number of time-matched measurements simultaneously recorded by the patients on the paper form as instructed. Causes of app errors and usability were also recorded.

## Methods

This was a single-cohort, open, prospective trial conducted at a French University Hospital (Hôpital Carémeau, CHU Nîmes, France). In accordance with the current French law and the Declaration of Helsinki, this study was approved by the institutional human investigation committee (Comité de Protection des Personnes, Sud Est V, Grenoble, France: 2017, A02790–53) and registered on ClinicalTrials.gov (NCT03464721; March 8, 2018) before starting [15].

Written informed consent was obtained from all participants before inclusion.

### Inclusion criteria

All patients > 18 years (ASA 1–3) scheduled for intermediate or major ambulatory surgery, with the ability to understand spoken and written French, were eligible and approached by the surgeon or the investigators. Surgeries were as follows: orthopaedic (shoulder repair, knee ligamentoplasty, hallux), abdominal (cholecystectomy, hernia) or gynaecological (hysterectomy, mastectomy).

### Exclusion criteria were

Age > 80 years, refusal to participate, ASA physical status > 3, emergency and inpatient surgery, psychiatric disorder.

Once included, patients were excluded if they failed to use the remote technology. The tablet and wireless Bluetooth monitor were presented by a nurse with the investigators and tested by the patient before surgery. Their ability to perform remote monitoring involved switching the tablet on, using the login, completing the self-report questionnaire, adapting the monitor (for MAP, HR and SpO^2^) and starting the monitor using the app (see above). Connection to a 4G network at home was also required.

### Intervention

- Routine ambulatory follow-up
  After surgery and before discharge from the ambulatory centre, patients received standard information regarding postoperative recovery and all the necessary information on postoperative care at home (analgesia, changes of dressing etc.). They were instructed to contact a 24-h telephone helpline should they have any questions or concerns outside office hours. Participants were advised to contact the local hospital’s emergency department should any emergency care be required.
- Smart Angel™ monitoring:
  The Smart Angel™ device was handed over to the patient who took the first measurements in presence of the team to ensure that the system was properly working and understood. Functionalities of the Smart Angel™ system were carefully explained and clear instructions for use were given by the research nurse and investigators, including how to move from question to question, how to enter an answer, and how to use the monitors. The Smart Angel™ system is a digital application using remote technology solutions and includes:

1. a web administration centre which collects, in real time, all the data for each patient transmitted by remote technology. These data are exported via the web (4G collection) to a secure server (Adista™, France). All data are then filtered and presented on a dashboard which summarizes them so that the nurses and/or physicians can focus on any warning signs in the patient. Each patient is depicted on the dashboard in the form of a coloured square (green: in the normal range = no problem; yellow: at the limit of range = but no sign of severity, red: warning signs = emergency action required). On the dashboard the investigators can see all patients enrolled in the study, their assessment and flags plus all the variables for each patient in a specific new window (see Fig. 1a, b, c).
2. Remote physical parameters (HR, SpO_2_, MAP) with ranges (min., max.) for normal values were defined before starting the study and included in the algorithm (Fig. 1c). In the event of a specific pathology or treatment (e.g. patient on beta-blockers), ranges can be adapted to specific patient characteristics and/or treatment.
3. The tablet and app technology uses a tablet (Samsung™, Korea) integrating the software (EvolucareLabs, France) which generates health questionnaires for the patient to answer with scores for pain relief, quality of recovery, nausea and vomiting (see above). This tablet communicates with a connected monitor at the patient’s home to perform discontinuous measurement. The app assesses the self-report questionnaires and the wireless Bluetooth monitors record the physiological measurements (heart rate, mean arterial blood pressure and blood oxygen saturation). The monitors used were: a wireless pulse oximeter clipped to the finger (iHeathlabs, USA) and a blood pressure monitor on the wrist (iHeathlabs, USA). When the patient is ready with the monitor, the software triggers the monitors and records the values. The patient can instantly see the tablet screen. Measurement data for each item is depicted with normal and abnormal ranges. All these data are then exported via the web (4G collection) to a secure server (Adista™, France).

- Follow-up:
  From the day of surgery to postoperative Day 5, three times a day (morning, noon and evening), the application prompts the patient to complete the health questionnaire and follow-up with the monitors. Seven variables are recorded for each assessment:
  1. Blood oxygen level (SpO^2^, %)
  2. Heart rate (HR, min^− 1^)
  3. Mean arterial blood pressure (MAP, mmHg)
  4. Vomiting (yes/no)
  5. Nausea (yes/no)
  6. Pain score (scored on an 11-point numerical pain scale; 0 = no pain, 10 = worst pain)
  7. Quality recovery score (scored on an 11-point numerical rating scale; 0 = poor condition, 10 = excellent quality of postoperative recovery)

At the end of our 5-day study, patient monitoring was stopped and the equipment was returned to the hospital by express delivery.

### Outcomes and data collection

Surgical, anaesthetic and patient characteristics data were collected by the research nurse and investigators. Seven variables (pain, quality of recovery, nausea, vomiting, HR, MAP and SpO^2^) were recorded by the app for all patients before their discharge from the centre and then three times a day from Day 1 to Day 5.

In addition, the number of time-matched variables simultaneously recorded by the patients on a paper form was returned to the centre at end of the study.

On Day 5, the patient had to answer a 10-item questionnaire with five response options ranging from “Strongly agree” to “Strongly disagree” (total: 1–100 points) and the result was converted into a System Usability Scale (SUS) based on a Lickert scale. The 10 items were:

- I think I will use the SMART ANGEL device frequently.
- I think the SMART ANGEL is unnecessarily complex.
- I find the SMART ANGEL easy-to-use.
- I think I will have to call technical support to be able to use this service.
- I find that the SMART ANGEL features are well integrated.
- I find that there are far too many inconsistencies in its use.
- I think most people will learn to use the SMART ANGEL device very quickly.
- I find the SMART ANGEL really heavy to use.
- I felt very confident using the SMART ANGEL.
- I had to learn a lot of things before I could use SMART ANGEL.

### Objectives

The primary objective of this pilot study was to compare the amount of data recorded on the website using the app with the paper form. Secondary objectives were to assess patient safety (medical rescue, readmission, and surgical complications) and the usability of this medical device.

### Sample-size calculation

As this was a pilot study, we had planned to test the system on 30 patients with no justification regarding sample-size. Published data has shown that 12 patients are a minimum requirement for pilot studies [16].

### Statistical analysis

Statistical analysis was conducted using SAS (9.4, SAS Inc., Cary NC).

Statistical results were expressed with mean (SD) or median with interquartiles [IQ] according to distribution. The numbers (with percentages, %) were given for categorical variables. The main judgment criterion was analysed in relation to a referential volume of theoretical information based on the following calculation: number of patients (*n* = 30) x number of data collection periods (i.e. one on Day 0 and 3 per day from Day 1 to Day 5, i.e. 16 in total) x number of parameters measured i.e. physiological parameters (heart rate, blood pressure, oxygen saturation and self-evaluation parameters (pain score, nausea, vomiting, comfort), i.e. 7 in total. Thus, the maximum reference volume of theoretical information is 30x16x7 = 3360. In addition, a referential volume of theoretical information was calculated per day and by parameter.

Comparisons of continuous variables between the app and paper questionnaire were made using a Student’s T-test or Wilcoxon-Mann-Whitney test according to distribution. Categorical variables were compared between groups (paper vs app data) by X^2^ or Fisher’s exact test. All *P* values were two-tailed and a *P*-value of less than 0.05 was required to exclude the null hypothesis. Analysis of secondary outcomes was descriptive.

## Results

### Population of the study, surgery and ambulatory setting

From May 2018 to September 2018, 30 patients were included and 29 analysed (1 patient was excluded as “ambulatory” as he was not discharged from hospital due to delayed surgery). Patients (15 male, 14 female) were 47 ± 13 years with a body mass index of 25 ± 3 kg.m^− 2^ and an ASA physical status 1/2 (*n* = 14/15). Surgery was either orthopaedic (*n* = 24, shoulder = 3, foot = 12, knee = 9) or abdominal (*n* = 5). Mean duration of surgery was 42 ± 21 min.

### Primary outcome

For 29 patients, 3248 (29x16x7) data items were to be collected on paper or by remote monitoring technology. The remote monitoring technology recorded 2038 data (62%) (Table 1).

**Table 1:** data recorded

	Theoretical	Paper	SmartAngel
Pain	464	390 (84)	297 (64)*
Comfort	464	394 (85)	297 (64) *
Nausea	464	394 (85)	297 (64) *
Vomiting	464	390 (85)	297 (64) *
Heart	464	370 (80)	297 (64) *
MAP	464	333 (72)	256 (55) *
SpO2	464	385 (83)	297 (64) *
Total	3248 (100)	2656 (81)	2038 (62)*

Results are number and percentage

*p < 0.05 compared to paper

**Fig. 2:**
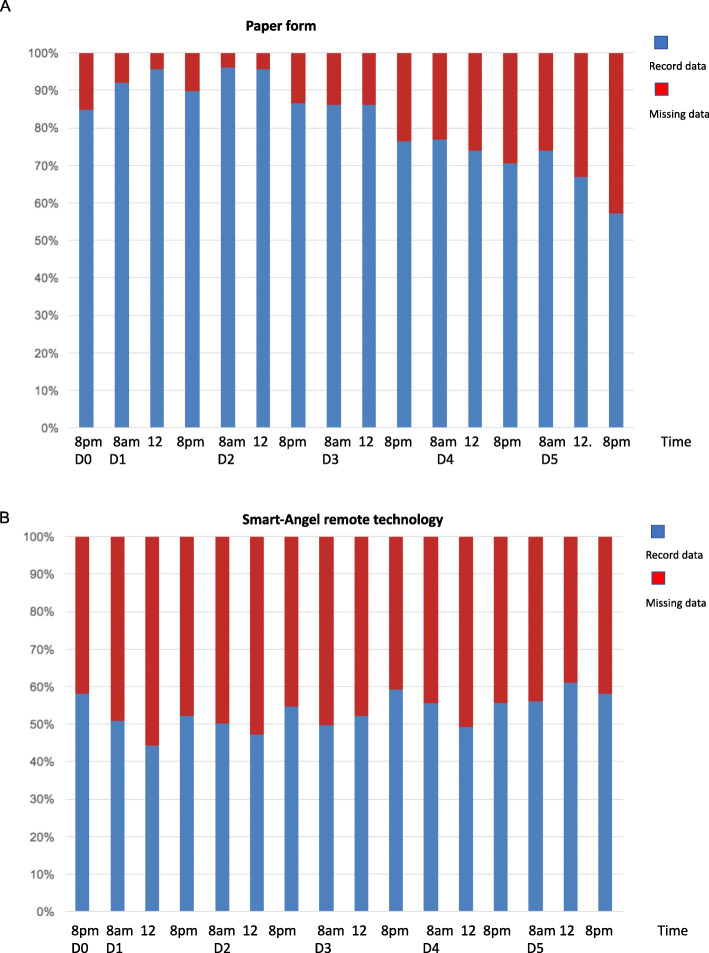
**a**: Patient’s written recordings. **b**: Patient’s remote monitoring

The conventional paper form recorded 2656 (82%) data. The difference between remote monitoring and paper form was statistically significant (*p* = 0.001) (Table 1). Figure 2a and b show the percentage of data recorded by patients per day at each time period on paper and via the web-solution, respectively.

### Secondary outcome

Concerning remote technology, three patients reported malfunctioning of the MAP monitor for all assessments from Day 0 to Day 5. The absent variables were later attributed to an internal software malfunction. On the eve of surgery, 12 (41%) patients reported difficulty to use the technology at home (forgotten password or login to starting using the tablet = 4, absence of 4G connection = 1, difficulty to transmit data from monitor to tablet = 7). On Days 1 and 2, 11 (39%) patients reported technical difficulties (4G connection = 1, difficulty to transmit data from monitor to tablet = 10). From Day 3 to Day 5, 9 (35%) patients reported similar difficulties.

With regard to usability, three patients did not perform the SUS survey. For the other 26 patients, the mean SUS score was 85 (68–93).

With regard to postoperative adverse effects during the first 5 days, the app recorded nausea in 7 patients and vomiting in one. These adverse events were consistent with the data noted by the patients on the paper forms. No patients were readmitted for adverse events during the study period.

On Day 30, 3 patients (10%) were readmitted for minor adverse outcomes (surgical complications), but none were due to the device.

## Discussion

In this first study reporting the use of a real-time remote monitoring device for outpatient surgery, the Smart Angel™ enabled patients to record > 60% of the required information. However, many technological failures were reported. These findings imply that real-time remote monitoring technology is feasible for outpatient surgery but still requires improvement, especially regarding connection to the central computer.

To our knowledge, the use of remote monitors for MAP, HR and SpO^2^ has never been evaluated in ambulatory surgery. Similar systems have been extensively tested and evaluated in cardiology, oncology and diabetes [17–20]. In these cases, they have contributed to optimising remote medical monitoring and adapting treatments [20]. In this sense, after ambulatory surgery, these monitors may be effective in detecting early postoperative adverse events in patients at home.

Out of 29 patients evaluated, 80% were able to visualize the values for MAP, HR and SpO^2^ on their monitors and could copy these values onto paper (Table 1). The originality of our study was to show that digital technology facilitates transmission of these data to a centre without any action from the patient (no recopying of data by the patient onto a smartphone or a web server). In this digital setting, the 62% data transmission rate by the SmartAngel™ device was disappointing. Apart from the fact that patients forgot to note their measurements, several technological reasons explain this lack of data feedback and our study has allowed us to better understand them.

1. An ineffective 4G environment at the patient’s home. The 4G defect altered data transmission between the tablet and the central web service in 3% of patients corresponding to the rates reported in teletransmission studies [16].
2. Malfunctioning of the SmartAngel™ program installed on the tablet. Computer program patches were required to stabilize the program for 3 patients as it had failed to activate the remote MAP monitors (confidential data provided upon request).
3. Insufficient battery charge for discontinuous but frequent use of the monitors. This is crucial for patients and they must be informed of the need to charge up the batteries regularly.

For the 62% of patients for whom all the data were correctly transmitted (Fig. 2b), the Smart Angel™ tool represented a truly original monitoring dashboard which had never been proposed before, providing questionnaire data combined with physiological data. The number of patients included was insufficient to demonstrate the interest of the system as an aid to follow-up, but the data presented are a step forwards in ambulatory follow-up and would be useful for a multicentric study.

Our study shows a decrease in the data collected on paper on Days 4 and 5 (Fig. 2a). This may suggest that optimal monitoring should be begun from the evening of Day 0 to the evening of Day 3 (with systematic measurements morning, noon and evening) and at least one measurement per day from Day 3 onwards to optimize patient adherence. Interestingly, the peak of postoperative complications classically occured between Day 1 and Day 3 [7].

In this study, the patient was monitored over the 5-day postoperative period without any manipulation from the expert centre. The high SUS score suggests good acceptance and usability by the patients [21]. Indeed, they willingly accepted the small-sized connected objects and the fact that there was a bag to transport all the objects home probably facilitated acceptance of the device. Several questions remains: the optimal time for instructing patients, the necessity to repeat instructions and the possibility of access to an on-line manual to help patient at home.

### Limitations of the study

The main limitations of the study are the monocentric design and the small number of patients included. However, the main objective of this first pilot study was to validate the concept and identify technological errors before carrying out a multicentric study. From this viewpoint, the present study provided the opportunity to report all potential issues with this technology, essentially, connection to the central computer, whatever the cause. Furthermore, we did not analyse whether age or intellectual level could influence the proper functioning of the system. Younger patients, who seem more likely to be tech-savvy, would have been able to troubleshoot issues with their iPads and bluetooth connections. However, it is the older patients who are more likely to have the kinds of operations where such remote real-time monitoring of vital signs and patient condition could be important or even life-saving. As these patients are also the ones who struggle the most with new technology, this would limit usability. Therefore this aspect needs to be evaluated.

## Conclusion

Future research is required to determine the exact role of remote non-invasive digital technology for delivering patient healthcare benefits and to evaluate the feasibility of large-scale implementation.

## Data Availability

The data that support the findings of this study are available from ^“^Department of Biostatistics, Epidemiology, Public Health and and Methodological innovation (BESPIM), Nîmes University Hospital, University Montpellier 1, France” but restrictions apply to the availability of these data, which were used under license for the current study, and so are not publicly available. Data are however available from the authors upon reasonable request and with permission.

## Abbreviations

Fig.: Figure
HR: Heart rate
MAP: Mean arterial blood pressure
PONV: Postoperative nausea vomiting
SpO_2_: Oxygen saturation
